# Predictive modeling of co-infection in lupus nephritis using multiple machine learning algorithms

**DOI:** 10.1038/s41598-024-59717-w

**Published:** 2024-04-22

**Authors:** Jiaqian Zhang, Bo Chen, Jiu Liu, Pengfei Chai, Hongjiang Liu, Yuehong Chen, Huan Liu, Geng Yin, Shengxiao Zhang, Caihong Wang, Qibing Xie

**Affiliations:** 1 Department of Rheumatology and Immunology, West China Hospital, Sichuan University, No. 37 Guo Xue Lane, Wuhou District, Chengdu, 610041 Sichuan China; 2 Department of Internal Medicine, Linfen People’s Hospital, Linfen, 041500 China; 3 School of Internet of Things, Jiangnan University, Wuxi, 214122 China; 4 Department of General Practice, General Practice Medical Center, West China Hospital, Sichuan University, Chengdu, China; 5 Department of Rheumatology, The Second Hospital of Shanxi Medical University, No. 382 Wu Yi Road, Taiyuan, 030001 Shanxi China

**Keywords:** Lupus nephritis, Infection, Machine learning, Lymphocyte subpopulations, Computational biology and bioinformatics, Immunology, Diseases, Nephrology, Rheumatology

## Abstract

This study aimed to analyze peripheral blood lymphocyte subsets in lupus nephritis (LN) patients and use machine learning (ML) methods to establish an effective algorithm for predicting co-infection in LN. This study included 111 non-infected LN patients, 72 infected LN patients, and 206 healthy controls (HCs). Patient information, infection characteristics, medication, and laboratory indexes were recorded. Eight ML methods were compared to establish a model through a training group and verify the results in a test group. We trained the ML models, including Logistic Regression, Decision Tree, K-Nearest Neighbors, Support Vector Machine, Multi-Layer Perceptron, Random Forest, Ada boost, Extreme Gradient Boosting (XGB), and further evaluated potential predictors of infection. Infected LN patients had significantly decreased levels of T, B, helper T, suppressor T, and natural killer cells compared to non-infected LN patients and HCs. The number of regulatory T cells (Tregs) in LN patients was significantly lower than in HCs, with infected patients having the lowest Tregs count. Among the ML algorithms, XGB demonstrated the highest accuracy and precision for predicting LN infections. The innate and adaptive immune systems are disrupted in LN patients, and monitoring lymphocyte subsets can help prevent and treat infections. The XGB algorithm was recommended for predicting co-infection in LN.

## Introduction

Lupus nephritis (LN) is a type of glomerulonephritis that is one of the most serious organ complications of the autoimmune disease systemic lupus erythematosus (SLE). It is characterized by hematuria, proteinuria, edema, hypertension, and renal insufficiency leading to functional impairment^1^. The pathogenesis of LN is caused by autoantibodies against nucleic acids in the body, deposition of immune complexes in the kidneys, extracellular trapping of neutrophils, and abnormal activation of the innate and adaptive immune systems^2^. Approximately 40–60% of SLE patients show clear clinical symptoms of LN^3^. Most SLE patients develop LN within five years of diagnosis, and in many cases, LN is also an important basis for SLE diagnosis. Active LN is closely related to overall morbidity and mortality in SLE patients^1,4^. The incidence of LN is 1–8.7 cases per 100,000 person-years, with a patient rate of 8–180 cases per 100,000 people^5,6^. Since LN is one of the multiple manifestations of SLE, many of the risk factors for LN overlap with those of SLE. Over the past few decades, there has been significant progress in understanding the genetics and pathogenesis of LN. Despite advances in the understanding of disease mechanisms and improvements in treatment options, LN remains a leading cause of morbidity and mortality in patients with SLE. Current treatments for LN include antimalarials, corticosteroids, and immunosuppressants, and more recently, target-specific biologic drugs have even been introduced^7^. Although these treatment options have improved LN-related outcomes^8,9^, LN is still associated with a higher occurrence of infection^10,11^. However, the development of new drugs to effectively manage LN activity while minimizing infection occurrence remains an ongoing challenge. Consequently, timely and precise diagnosis of LN infection, coupled with prompt treatment, is of paramount importance in order to enhance the prognosis of LN patients.

Machine learning (ML) is rapidly emerging in the medical field and is expected to revolutionize clinical practice in the foreseeable future^12,13^. ML is a complex computational process that uses mathematical models and training data to gain predictive power. ML methods play an important role in building appropriate models to discover potential correlations and future predictions by learning and evaluating data patterns. Rather than explicitly deriving results from predetermined rules, ML unlocks the potential to excel at tasks such as identifying and analyzing complex data patterns by deriving parameters from instances^14^. Research has demonstrated that ML techniques can effectively discern immune patterns linked to various subtypes of juvenile idiopathic arthritis^15^ and can also identify clusters of long-term autoantibody profiles that can predict disease outcomes in SLE^16^. In addition, research also shows that it is possible to build a model that predicts 1-year treatment response in patients with LN using new ML methods^17^. At present, no corresponding model has been specifically established to predict the co-infection of LN patients, and the prediction performance of existing models cannot meet clinical needs. Therefore, this study aimed to investigate the applicability of ML in predicting the presence of infections in patients with LN. A diverse range of machine learning algorithms were employed, and the dataset was split into a training group and a testing group. A total of 183 patients were randomly allocated to either the training group or the test group. Following adequate training of the predictive model, validation was carried out using the test group.

## Methods

### Recruitment of participants

From the electronic medical records system of the Rheumatology and Immunology Department at the Second Hospital of Shanxi Medical University, we retrieved data on lupus nephritis patients who were treated between July 2015 and November 2016. Basic information about study subjects and initial laboratory test results for all patients were collected into the medical record system within 72 h of admission. Experienced rheumatologists screened eligible patients according to study criteria and meticulously reviewed their medical records. A total of 183 patients were analyzed, including 160 females and 23 males. During data collection, detailed records of patients’ demographics, clinical symptoms, laboratory test results, sites of infection, medication history, etc., were documented by physicians (Supplementary Table 1). If any examination results were missing from patients' records, research assistants contacted the hospital laboratory to retrieve the data, ensuring data integrity and accuracy. All patients met the American College of Rheumatology (ACR) and European College of Rheumatology/European Association for the Research of Renal Diseases (EULAR/ERA-EDTA) guideline criteria^18,19^. Additionally, we recruited 206 healthy individuals from the Physical Examination Center of the Second Hospital, matched by age and sex, to serve as the healthy control (HCs) group. A rheumatology immunologist with extensive clinical experience recorded detailed patient information, including clinical data, infection site, medication status, etc. The exclusion criteria for the study included patients with incomplete clinical data (missing data for characteristics account for more than 20% of the total sample), individuals under the age of 18, patients diagnosed with other connective tissue diseases, individuals with malignant tumors, immunodeficiency, or severe cardiopulmonary insufficiency, patients with a history of drug allergies or mental illness, patients who have recently undergone digestive endoscopy or surgery, and pregnant or lactating women. All participants in the study provided their informed consent, and the Clinical Research Ethics Committee of the Second Hospital of Shanxi Medical College (Taiyuan, China, 2017-KY-004) approved the study. All methods were performed in accordance with the relevant guidelines.

### Infection defined

We use various methods to determine whether an infectious disease is caused by bacteria or viruses. These methods include reviewing the patient's medical history, conducting a physical examination, and performing ancillary examinations. We confirm the presence of infection through positive pathogen tests or by identifying conclusive evidence of infection, such as an abscess found in a computed tomography scan, based on various specimens like blood, sputum, pus, stool, and urine. Furthermore, we consider a fever (body temperature exceeding 38.0 °C) as an infection if it lasts for at least 3 days and is effectively reversed after anti-infective treatment. However, we do not record the presence of infection if there is no evidence to support it, or if there is doubt about the cause of current symptoms.

### The method of model establishment

We used the Python programming language (Python Software Foundation, version 3.6) for data analysis. During the analysis process, we used 8 ML algorithms and used the training group to build corresponding models, and then verified the results in the test group. Select variables for predicting LN infection in the training group and train ML models, including logistic regression (LR), decision tree (DT), k-nearest neighbor (KNN), support vector machine (SVM), Multilayer Perceptron (MLP), Random Forest (RF), Ada Boosting (Ada) and Extreme Gradient Boosting (XGB) (Supplementary Table 2). Initially, the independent variables were standardized to ensure they were measured on a consistent scale, while missing data were imputed through multiple imputation techniques. Additionally, we manually tuned the parameters of each model. The samples are randomly divided into training group and test group, model training is performed on the training group, and model verification is performed on the test group (Supplementary Tables 3 and 4). To select a subset of features to obtain the smallest size and optimal performance, we employ the Random Forest-based Sequential Forward Selection algorithm. The algorithm evaluates model performance (F1_score) by adding one feature at a time to a subset of features and iteratively generating a new model. F1_score is a comprehensive evaluation index of precision and recall. A higher F1_score signifies greater robustness of the model. When the F1_score of the feature subset reaches the optimal value, the iteration is stopped and the feature subset with the smallest size and optimal performance is selected. The Scikit-learn package (Scikit Learning (https://github.com/scikit-learn/scikit-learn)) was used for ML^20^. The data processing and model establishment workflow is visually presented in Supplementary Fig. 1.

### Statistical analysis and model evaluation

To evaluate the prediction model, we used the confusion matrix performance metric to measure the effectiveness of the model and visualized the confusion matrix through the Matplotlib package. To evaluate the performance of the prediction model, we compared multiple evaluation metrics, including the area under the receiver operating characteristic (ROC) curve (AUC), accuracy, recall, precision, and F1 score. The specific evaluation index formula is shown in Supplementary Table 5. An effective model should achieve good performance on both the training group and the test group. The closer the ROC curve is to the upper left corner, the more representative the model is, that is, the AUC is close to 1. Finally, through comprehensive performance comparison of these evaluation criteria, we identified the best model for predicting LN co-infection or not. Statistical analysis used SPSS 26.0 software. The categorical demographic characteristics of patients were compared using the χ test. When continuous data satisfy normality and homogeneity of variances, they are expressed as mean (± standard deviation). The independent sample *t*-test was employed to compare two groups, while one-way analysis of variance (ANOVA) was used to compare multiple groups. For data that met normality or homogeneity of variance, the median (range) was used to express the data, and the Mann–Whitney *U* test was used for comparison between groups. Correlation analysis used spearman correlation test. All statistical tests were conducted by bilateral test, and P < 0.05 was considered statistically significant.

### Ethics statement

The studies involving human participants were reviewed and approved by the Clinical Research Ethics Committee at the Second Hospital of Shanxi Medical College and West China Hospital College. All methods were performed in accordance with the relevant guidelines.

## Results

### Analysis of baseline information

Among these 183 patients, 160 were female (87.4%), and infection occurred in 111 patients (32.7%). There was no statistically significant difference in sex between infected and non-infected patients (P > 0.05). Compared with patients with noninfectious lupus nephritis, patients with infectious LN had lower levels of red blood cells, hemoglobin, platelets, and lymphocytes (P < 0.01), but a higher proportion of erythrocyte sedimentation rate (ESR) and C-reactive protein (CRP) (Table 1). All patients received conventional glucocorticoid and immunosuppressive treatment at baseline, including hydroxychloroquine (78.14%), tacrolimus (13.66%), methotrexate (7.1%), leflunomide (15.85%), cyclophosphamide (61.75%), mycophenolate mofetil (24.59%). In terms of the site of infection, respiratory tract infections prevail as the most prevalent (69.4%), encompassing herpes zoster, upper respiratory tract infections, nasopharyngitis, and bronchitis. Subsequently, gastrointestinal infections (20.8%) and urinary tract infections (12.5%) ensue as the next categories (Supplementary Table 6). Concerning pathogens, bacterial infection was more common. Viral infections include Epstein–Barr virus, cytomegalovirus, and respiratory syncytial virus.

**Table 1:** Comparison of basic information and clinical laboratory characteristics between non-infected and infected patients with LN during hospitalization.

Characteristics	Non-infected (n = 111)	Infected (n = 72)	*P* value
Age, year, mean ± SD	36.84 ± 13.62	36.85 ± 14.23	1.00
Female, no. (%)	96 (86.49)	64 (88.89)	0.63
Duration, month, median (range)	83.56(0.36)	84.30(1.48)	0.96
WBC, × 10^9^/L, mean ± SD	6.20 ± 3.60	5.53 ± 3.52	0.22
RBC, × 10^12^/L, mean ± SD	3.87 ± 0.66	3.51 ± 0.81	0.001**
HB, g/L, mean ± SD	111.69 ± 22.13	98.88 ± 23.05	< 0.001***
PLT, × 10^12^/L, mean ± SD	189.94 ± 82.09	152.62 ± 75.26	0.002**
LYMP, × 10^9^/L, mean ± SD	1.34 ± 0.67	0.98 ± 0.59	< 0.001***
NEUT, × 10^9^/L, mean ± SD	4.59 ± 3.61	4.09 ± 3.14	0.34
ESR, mm/h, mean ± SD,	45.58 ± 37.32	56.73 ± 37.47	0.05
CRP, mg/dL, mean ± SD,	6.56 ± 11.30	10.29 ± 15.84	0.11
Prednisone dose, mg/day, median (range)	36.15 (0.60)	36.97 (0.60)	0.80
Use of concomitant agents (no. of patients)			
Hydroxychloroquine	88	55	–
Tacrolimus	18	7	–
Methotrexate	10	3	–
Leflunomide	21	8	–
Cyclophosphamide	75	38	–
Mycophenolate mofetil	29	16	–

*LN* lupus nephritis, *WBC* white blood cell, *RBC* red blood cell, *HB* hemoglobin, *PLT* platelet, *LYMP* lymphocyte, *NEUT* neutrophil, *ESR* erythrocyte sedimentation rate, *CRP* C-reactive protein. *P < 0.05; **P < 0.01; ***P < 0.001.

### Comparison of peripheral lymphocyte subsets among LN infected group, non-infected group, and healthy controls

**Figure 1:**
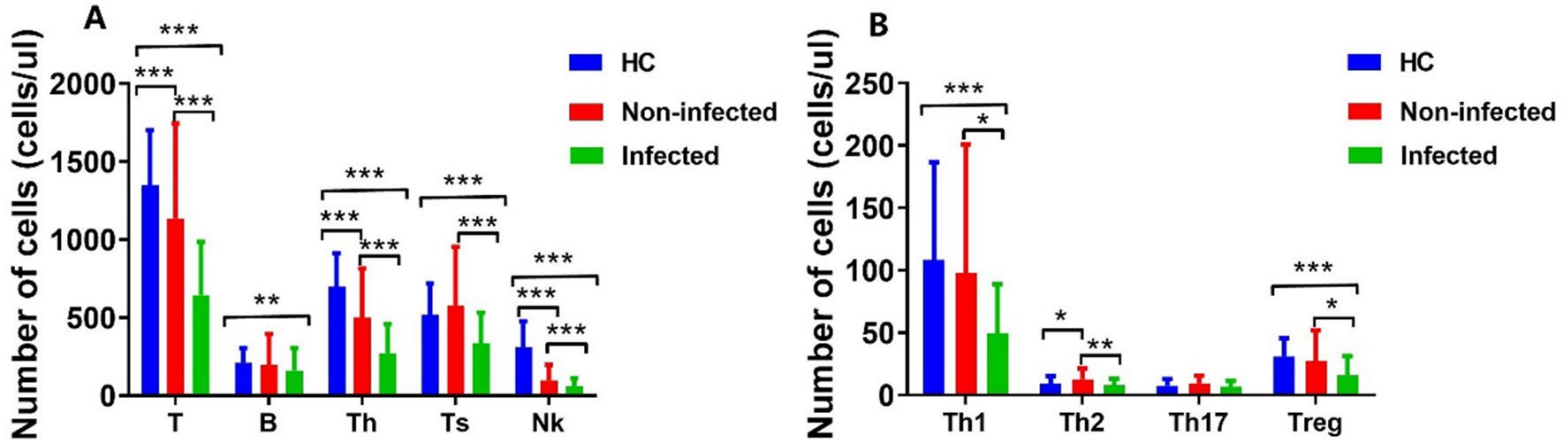
Changes in peripheral lymphocyte subsets in LN infected group, non-infected group, and healthy control group. Data were presented as mean ± SD and statistical analysis was determined by a two-tailed paired *t*-test. *P < 0.05, **P < 0.01, ***P < 0.001. *LN* lupus nephritis, *HC* healthy control, *Th* helper T-cells, *Ts* suppressor T cell, *NK* natural killer cell, *Treg* regulatory T cells.

The absolute numbers of T, helper T cells (Th), and natural killer cells (NK) cells in the non-infected group were significantly lower than those in the HCs (P < 0.001), but were still dramatically higher than those in infected patients (P < 0.001). Compared with HCs, patients in the infected group had significantly lower levels of NK, B and suppressor T cells (Ts) cells (P < 0. 01), while there was no significant difference in these cells between health controls and the non-infected patients (P > 0.05) (Fig. 1A and Supplementary Tables 7 and 8). In terms of Th cell subsets, the infection group had the lowest levels of Th1 and Treg cells (P < 0.05), while the difference between the non-infection group and the HCs was not statistically significant (P > 0.05). The non-infected group exhibited a notably elevated level of Th2 cells compared to the HCs (P < 0.05). Additionally, the absolute numbers of infected patients were significantly lower than those in the non-infected groups (P < 0.001) (Fig. 1B and Supplementary Tables 7 and 8).

### Feature analysis and evaluation of predictive value of regression model

**Figure 2:**
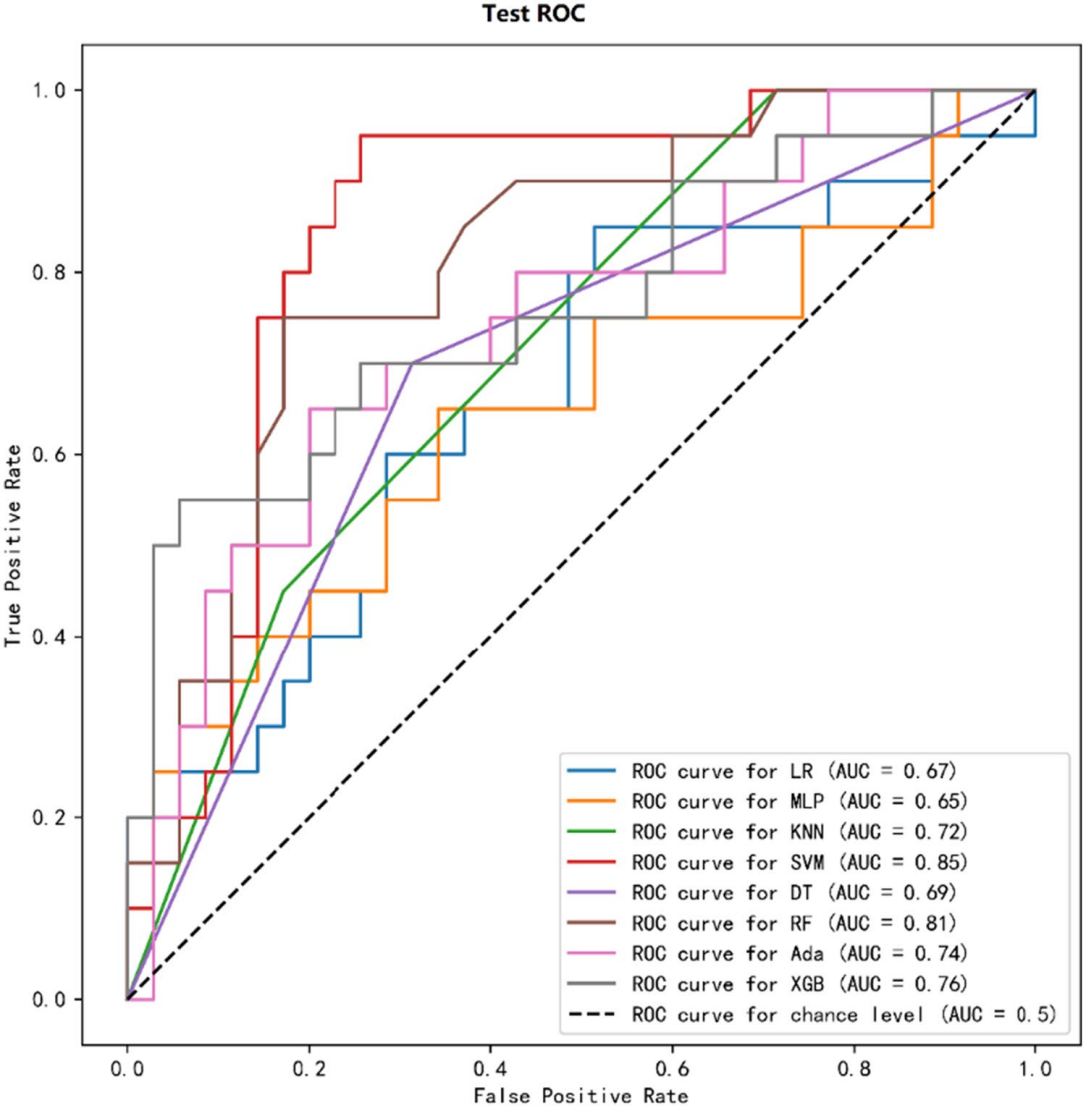
The machine learning algorithm predicts the AUC value of lupus nephritis infection in the test group. *LR* logistic regression, *DT* decision tree, *KNN* K-nearest neighbors, *SVM* support vector machine, *MLP* multi-layer perceptron, *RF* random forest, *Ada* Ada boost, *XGB* extreme gradient boosting, *ROC* receiver operating characteristic, *AUC* area under the curve.

The correlation of each variable with LN infection can be determined through the heat map (Supplementary Fig. 2). The XGB algorithm suggests that T, red blood cell count (RBC), length of hospitalization (LOS), and lymphocyte count (LYMP)% are the four most important weights for infection identified of LN (Supplementary Table 9). While, the top 6 weighting factors for infection identified by the SVM in ML were Th%, RBC, T, LOS, white blood cell count (WBC), and CRP (Supplementary Table 10). In addition, the most important influencing factor in the Ada, RF, and LR algorithms was Th%, T, and Th% respectively (Supplementary Tables 11–13). In each algorithm model, the influencing factor of age was higher than that of sex, and sex had little relationship with infection of LN. During the training process, we randomly divided the original data into a training group and a test group in a ratio of 7:3. Given the imbalanced ratio between the infected and non-infected groups (positive and negative samples) in the data, we employed various random states to ensure that the distribution of positive and negative samples in the training and test groups aligns with the original dataset’s distribution. It is determined that random state = 1024, the positive and negative sample distribution ratio of each data set (original data set, training group, and test group) have the best effect (Supplementary Fig. 4). We randomly divided 183 patients into a training group (N = 128) and a testing group (N = 55). Confusion matrix for different ML models were shown in Supplementary Table 5. Comparison of basic characteristics of patients in the training group and the test group: there was no statistically significant difference in age (P = 0.837) and sex (P = 0.659) between the two groups (Supplementary Table 14). In the training group, the AUC values and accuracy and precision of DT, RF, Ada, and XGB were 1.00, 100%, and 100%, respectively. The AUC values and accuracy and precision of MLP were 0.98, 94.53%, and 92.45%, respectively (Supplementary Fig. 5 and Supplementary Table 15). The model was further performed to predict infection in the experimental group. Among the models constructed by the 8 algorithms, the four algorithms with the highest AUC values were SVM, RF, XGB, and Ada; their values were 0.85, 0.81, 0.76, and 0.74, respectively. Taking these impressive results into consideration, the XGB algorithm demonstrates superior performance with an accuracy of 80% and the highest precision rate of 84.62% (Fig. 2 and Table 2).

**Table 2:** Performance of machine learning algorithm predicts co-infection in test groups.

Test model name	AUC	Accuracy (%)	Precision (%)	Recall (%)	F1
LR	0.67	63.64	50.00	35.00	0.4118
MLP	0.65	63.64	50.00	50.00	0.5
KNN	0.72	69.09	60.00	45.00	0.5143
SVM	0.85	72.73	66.67	50.00	0.5714
DT	0.69	69.09	56.00	70.00	0.6222
RF	0.81	72.73	69.23	45.00	0.5455
Ada	0.74	70.91	62.50	50.00	0.5556
XGB	0.76	80.00	84.62	55.00	0.6667

*LR* logistic regression, *DT* decision tree, *KNN* K-nearest neighbors, *SVM* support vector machine, *MLP* multi-layer perceptron, *RF* random forest, *Ada* Ada boost, *XGB* extreme gradient boosting, *AUC* area under the curve.

## Discussion

Possible causes of infection include lymphocyte disorders and immune system abnormalities^21,22^. Our study shows that LN patients have lower levels of T, B, NK, Th, Ts, Th1, Th2, and Treg cells in the peripheral blood. The decrease in these cells was even more pronounced in patients with LN co-infection. It can be seen that both the innate immunity and the adaptive immune system of LN patients are disordered, and monitoring the levels of lymphocyte subpopulations in LN patients may help prevent and control infections. Activated naive CD4+ T cells can differentiate into Th cells, including Th1, Th2, and Th17, and Treg cells^23^, which are closely related to the development of SLE^24^. T cells are also the main component of infiltrating LN, and their phenotype is an exhausted state^25^. In our study, coinfected LN patients had the lowest numbers of Th1 and Treg cells. Interestingly, the absolute cell number in the infected group was lower than that in the non-infected group, while the levels of Ts and Th2 cells in the non-infected group were higher than those in the HCs. The increase in Ts and Th2 cells in the non-infected group may mean sustained immune activation or susceptibility to allergic or inflammatory responses.

LN is one of the most common and severe complications of SLE, especially in non-Caucasian patients, and it is reported that the cumulative incidence rate in Chinese SLE patients can reach 60% within 5 years of SLE diagnosis^26^. LN is usually treated with immunosuppressive agents such as glucocorticoids, cyclophosphamide, or mycophenolate mofetil. Although the application of hormones, immunosuppressants and biological agents is beneficial for the treatment of LN, there is also a potential risk of infection^27^. Epidemiological studies have shown that infection is the second most common cause of death in patients with LN-related chronic kidney disease^28,29^. The prevalence of infection increases with age and disease duration. For LN patients with active disease, the possibility of co-infection should be particularly vigilant. Our data confirm that LN coinfection can occur at multiple sites. Among them, respiratory tract infections are the most common, followed by gastrointestinal and urinary tract infections, which is consistent with previous research results^27^. LN patients themselves have a high occurrence of infection, and infection can induce an exacerbation of LN disease. Clinically, it is necessary to clarify the influencing factors of co-infection in LN patients. Establishing a preliminary co-infection assessment model based on ML may help early diagnosis of LN patients with co-infections, allowing for active intervention and effective treatment. The onset and infection of LN are associated with poor long-term renal prognosis in SLE patients. Hence, to effectively manage LN patients' disease progression and minimize hospitalization duration, it is imperative to closely monitor the patients' clinical and laboratory indicators, judiciously administer glucocorticoids and immunosuppressants, enhance their immune function, and actively regulate the condition of LN.

The training group is used to train the supervision model, fit the model, and adjust parameters to select the best algorithm; while the test group is used to evaluate the effect of the trained model without changing the parameters and effects of the model. We evaluated the output of eight ML models and compared their accuracy in predicting LN co-infection via clinical and numerical performance metrics. The research results show that the XGB model shows good performance in predicting the LN co-infection or not. Furthermore, based on the outcomes generated by the XGB model, the most significant factors contributing to infection, in descending order of importance, are T, RBC, LOS, and LYMP%. Previous studies usually used classical regression methods to identify risk factors and build risk prediction models^30^. However, these methods may not capture nonlinear relationships between explanatory and outcome variables. In contrast, ML techniques pay more attention to the deviation between predicted values and actual values and are better able to handle these limitations. ML methods also take into account more information gain, naturally eliminate linear correlations, and avoid non-linear correlations. Predicting LN co-infection by ML methods based on performance predictors is feasible and was evaluated based on clinical data sets in this study.

Our study is the first to use multiple ML algorithms to predict whether LN is coinfected. This study uses ML methods to build a co-infection prediction model that can deeply mine data based on real-world evidence. Through an extensive comparison of various algorithms, we have determined that the XGB model exhibits the most robust predictive capabilities among them. To the best of our knowledge, there are currently few studies on LN co-infection prediction models. This study provides new perspectives and guidance for LN, making the infection model more concise and accurate. In contrast to traditional models, ML models possess the ability to uncover and leverage untapped variables, effectively mitigating the limitations inherent in real-world clinical experience. Nonetheless, it is essential to acknowledge and address several limitations in our work. First, this was a retrospective study design, and the recorded data were irregular or incomplete, preventing us from incorporating new variables. Therefore, we make it more balanced by removing as much noise and imperfect records as possible from the dataset. Secondly, this study has only been internally validated and has not yet been externally validated, requiring prospective cohort validation from more centers in the future. Third, feature extraction and screening have a great impact on research results. Finally, the limited sample size in this study limits the possibility of further optimizing the performance of the model. In the future, our model's performance is poised to further enhance as we expand our scope by testing diverse classification techniques on larger, multicenter, qualitative datasets.

## Conclusion

We conducted infection prediction of LN using eight ML algorithms. Our research found that the XGB algorithm outperformed other models in terms of prediction accuracy, which may be one of the preferred options for studying patients with co-infected LN. Clinicians can utilize the XGB algorithm to early and effectively identify individuals LN with infection or not.

### Supplementary Information

Supplementary Figures.

Supplementary Tables.

## Data Availability

The original data supporting the conclusions of this article will be made available without reservation by the authors. Further inquiries can be directed to the corresponding authors.

## Abbreviations

ML: Machine learning
SLE: Systemic lupus erythematosus
LN: Lupus nephritis
HC: Healthy control
ESR: Erythrocyte sedimentation rate
CRP: C-reactive protein
PBMC: Peripheral blood mononuclear cells
FCM: Flow cytometry
CI: Confidence interval
ROC: Receiver operating characteristic
AUC: Area under the curve
LOS: Hospital length of stay
WBC: White blood cell
RBC: Red blood cell
LYMP: Lymphocyte
NK: Natural killer cell
Th: Helper T-cells
Ts: Suppressor T cell
Treg: Regulatory T cells
LR: Logistic regression
DT: Decision tree
KNN: K-nearest neighbors
SVM: Support vector machine
MLP: Multi-layer perceptron
RF: Random forest
Ada: Ada boost
XGB: Extreme gradient boosting
TP: True positive
TN: True negative
FP: False positive
FN: False negative
