# Supplementary material for: Predictive modeling of co-infection in lupus nephritis using multiple machine learning algorithms
Source: Sci Rep. 2024 Apr 22;14:9242. doi: 10.1038/s41598-024-59717-w (PMC11035552; doi:10.1038/s41598-024-59717-w)
Supplement: Supplementary file 1 — Supplementary Figures. [file 41598_2024_59717_MOESM1_ESM.docx]

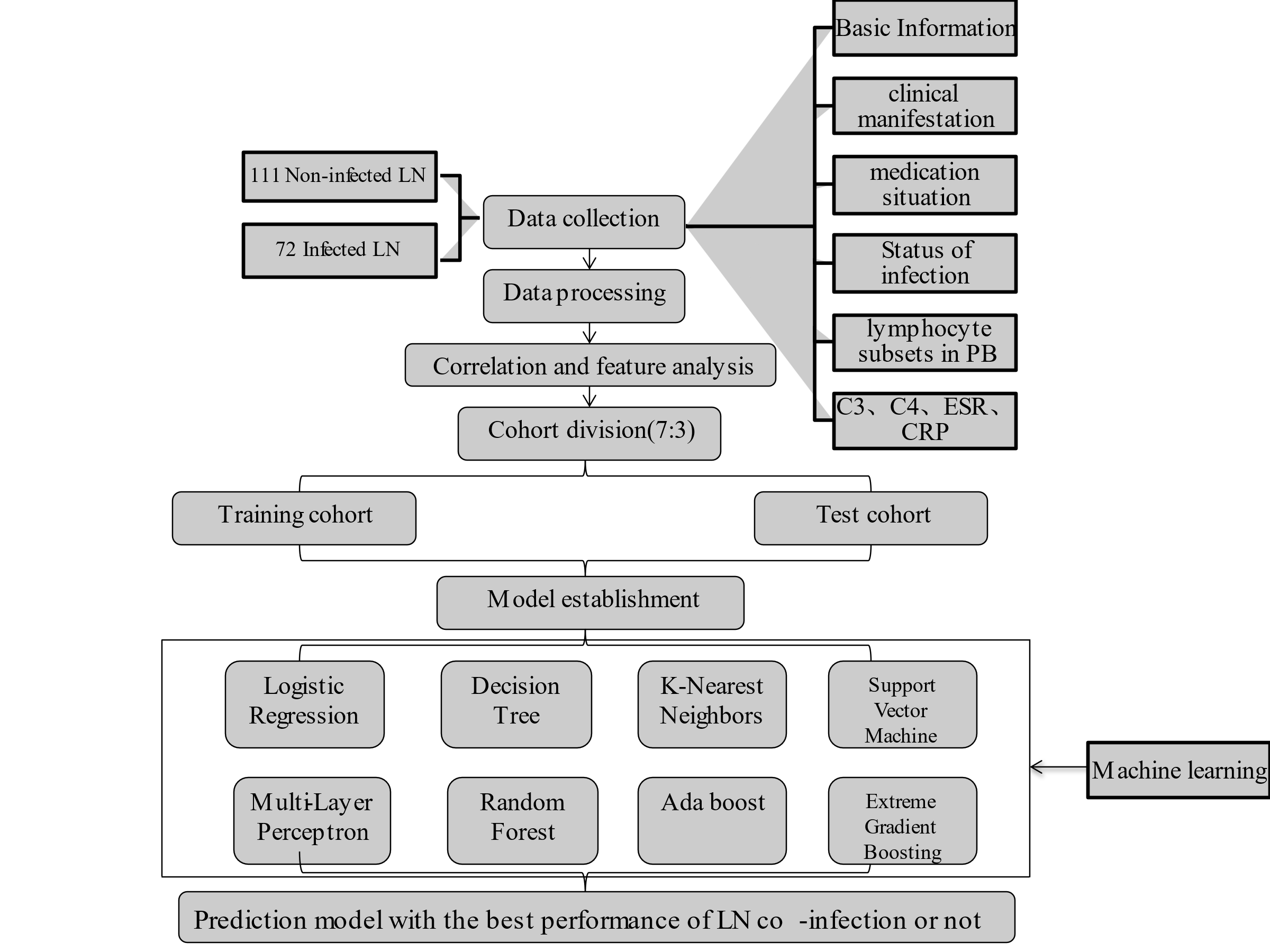


**Supplementary Figure 1:** The workflow of data processing and model establishment. LN, lupus nephritis; PB, peripheral blood


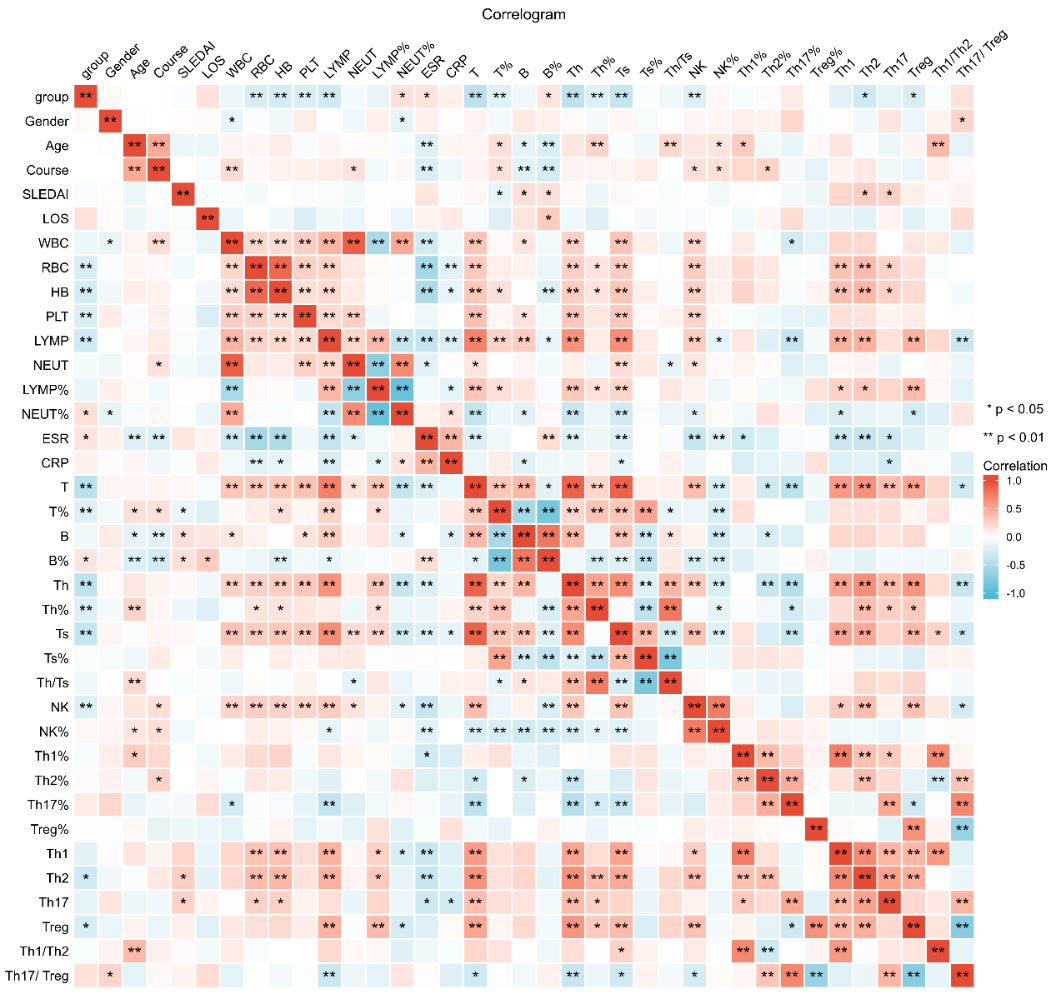


**Supplementary Figure 2**. Correlation between individual variables and lupus nephritis with infection. Group (Non-infection, Infection). SLEDAI, Systemic lupus erythematosus disease activity index. LOS, hospital length of stay. WBC, white blood cell. RBC, red blood cell. HB, hemoglobin. PLT, Platelet. LYMP, lymphocyte. NEUT, neutrophil. ESR, erythrocyte sedimentation rate. CRP, C-reactive protein. NK, Natural killer cell. Th, helper T-cells. Ts, suppressor T cell. Treg, regulatory T cells. The depth of the color block represents the magnitude of the correlation coefficient, *P < 0. 05, **P < 0. 01, *** P < 0. 001.


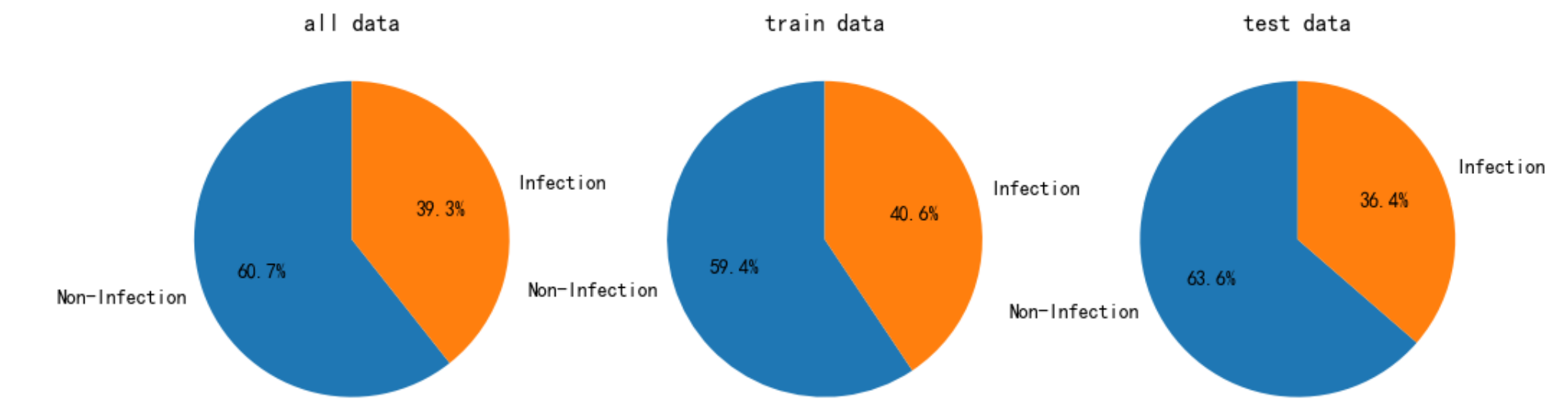


**Supplementary Figure 4**. Proportion of sample distribution of infected and non-infected groups for all original datasets, training, and test sets.


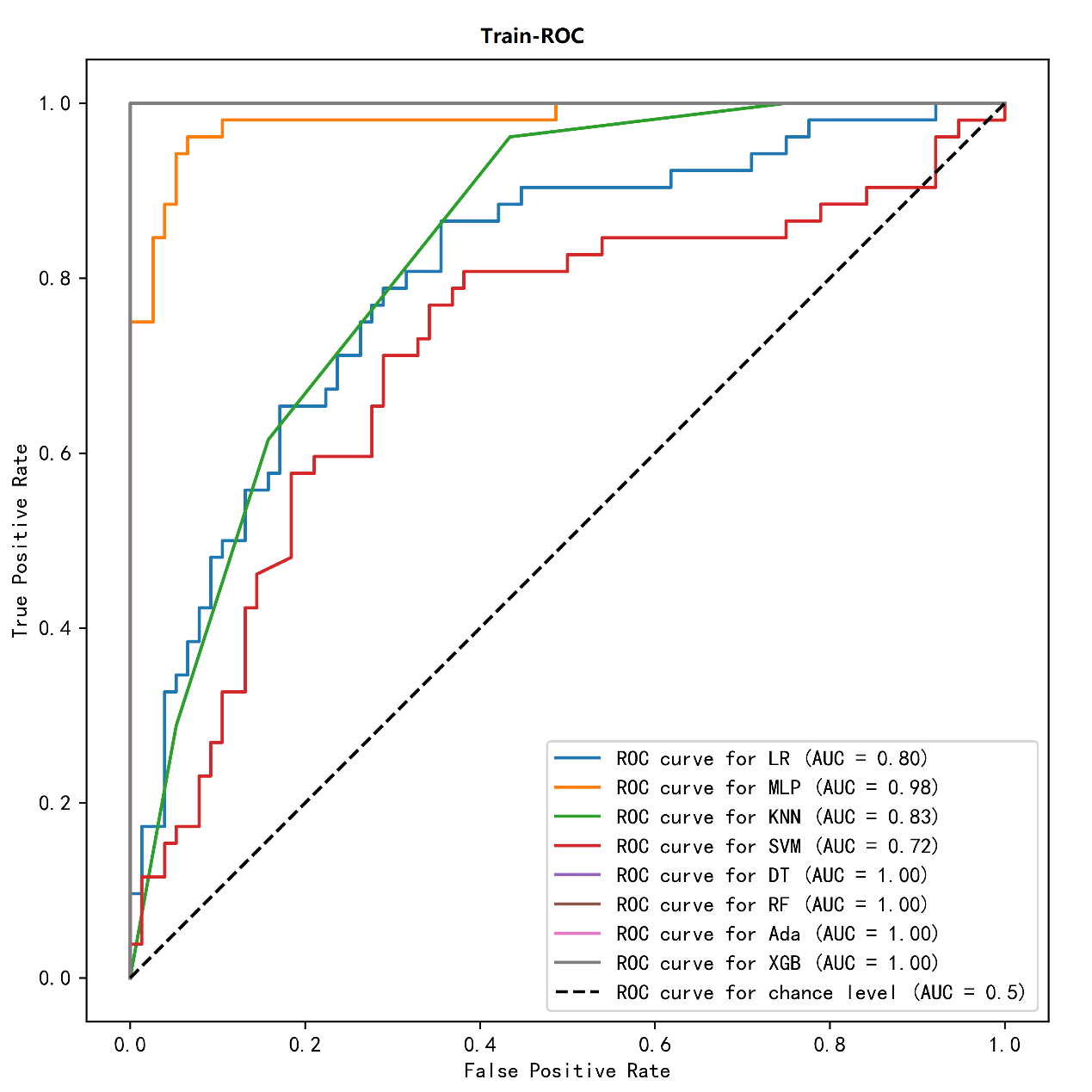


**Supplementary Figure 5**. The artificial intelligence algorithm predicts the AUC value of lupus nephritis infection in the training group. LR, Logistic Regression; DT, Decision Tree; KNN, K-Nearest Neighbors; SVM, Support Vector Machine; MLP, Multi-Layer Perceptron; RF, Random Forest; Ada, Adaboost; XGB, Extreme Gradient Boosting; ROC, Receiver Operating Characteristic; AUC, Area Under the Curve.
